# Emotional Stress and Cardiovascular Complications in Animal Models: A Review of the Influence of Stress Type

**DOI:** 10.3389/fphys.2016.00251

**Published:** 2016-06-24

**Authors:** Carlos C. Crestani

**Affiliations:** Faculdade de Ciências Farmacêuticas, UNESP - Univ Estadual PaulistaAraraquara, Brasil

**Keywords:** psychological stress, restraint stress, chronic variable stress, social isolation, social defeat, crowding stress, baroreflex, autonomic activity

## Abstract

Emotional stress has been recognized as a modifiable risk factor for cardiovascular diseases. The impact of stress on physiological and psychological processes is determined by characteristics of the stress stimulus. For example, distinct responses are induced by acute vs. chronic aversive stimuli. Additionally, the magnitude of stress responses has been reported to be inversely related to the degree of predictability of the aversive stimulus. Therefore, the purpose of the present review was to discuss experimental research in animal models describing the influence of stressor stimulus characteristics, such as chronicity and predictability, in cardiovascular dysfunctions induced by emotional stress. Regarding chronicity, the importance of cardiovascular and autonomic adjustments during acute stress sessions and cardiovascular consequences of frequent stress response activation during repeated exposure to aversive threats (i.e., chronic stress) is discussed. Evidence of the cardiovascular and autonomic changes induced by chronic stressors involving daily exposure to the same stressor (predictable) vs. different stressors (unpredictable) is reviewed and discussed in terms of the impact of predictability in cardiovascular dysfunctions induced by stress.

## Introduction

Convergent clinical and preclinical studies have provided evidence on the important role of psychosocial factors in the etiology and progression of cardiovascular diseases (Friedman and Rosenman, 1959; Rosenman et al., 1975; Kawachi et al., 1994; Ford et al., 1998; Rozanski et al., 1999; Rugulies, 2002; Smith et al., 2004; Grippo and Johnson, 2009; Roest et al., 2010; Carnevali et al., 2013a; Sgoifo et al., 2014). Among several psychosocial factors, psychological stress has been recognized as a modifiable risk factor for several cardiovascular dysfunctions (Steptoe and Kivimaki, 2012; von Känel, 2012; Inoue, 2014). Indeed, epidemiological and experimental results on humans and animals have demonstrated the influence of psychosocial stress on cardiovascular health (Rosengren et al., 2004; Kivimaki et al., 2006b; Grippo and Johnson, 2009; Steptoe and Kivimaki, 2012; Jarczok et al., 2013; Sgoifo et al., 2014). The association between stress and cardiovascular diseases has been shown to be independent of traditional cardiovascular risk factors (e.g., age, sex, smoking, diabetes mellitus, and obesity; Rosengren et al., 2004; Kivimaki et al., 2006b; Steptoe and Kivimaki, 2012).

The impact of stress on physiological and psychological processes is determined by stressor characteristics, such as chronicity, predictability, and severity (Nalivaiko, 2011; Steptoe and Kivimaki, 2012; Herman, 2013). Indeed, distinct cardiovascular and neuroendocrine changes have been reported following acute vs. chronic stress regimens (Nalivaiko, 2011; Herman, 2013). Furthermore, studies in animals comparing the impact of predictable vs. unpredictable stressor stimuli demonstrated that the latter induces more severe behavioral and physiological consequences (Bassett et al., 1973; Magariños and McEwen, 1995; Marin et al., 2007; Flak et al., 2012; Kopp et al., 2013; Smith et al., 2013; Duarte et al., 2015a). A dose–response pattern of stress-induced cardiovascular dysfunctions has also been reported, and stress severity may be modulated by the duration and/or frequency of the exposure to stress stimulus (Marmot et al., 1991; Kivimaki et al., 2006a; Chandola et al., 2008; Steptoe and Kivimaki, 2012).

The purpose of the present review is to discuss experimental research in animal models describing the impact of emotional stress on cardiovascular function. The primary focus of this review is to discuss the influence of stress type in stress-induced cardiovascular changes. Therefore, the first section discusses the influence of chronicity by summarizing the cardiovascular responses during acute stress sessions and the cardiovascular dysfunctions following repeated exposure to aversive stimuli (i.e., chronic stress). The second section discusses the impact of the predictability of stressor stimulus in cardiovascular and autonomic changes induced by chronic stress.

## Cardiovascular responses to stress: influence of chronicity

### Cardiovascular responses during acute stress sessions

A coordinated and complex set of physiological changes is generated during acute stress sessions. Changes in the autonomic nervous system activity promote immediate responses during aversive threats, which are mainly characterized by changes in the cardiovascular function. Cardiovascular responses typically consist of increase in blood pressure, heart rate (HR), and cardiac output (Hubbard et al., 1986; Schadt and Hasser, 1998; Dos Reis et al., 2014). However, these responses are consequences of more complex changes in cardiovascular function. Below is discussed the blood flow redistribution, changes of cardiac autonomic activity, and modulation of baroreflex function observed during an acute stress session. Additionally, the recovery of cardiovascular function after ending the stress session is described.

#### Blood flow redistribution

Blood flow redistribution occurs from the visceral and cutaneous beds toward the skeletal muscle vasculature during aversive threats (Kirby et al., 1987; Kapusta et al., 1989; Knardahl and Hendley, 1990; Viken et al., 1991; Zhang et al., 1996; Schadt and Hasser, 1998; Blessing, 2003). Blood flow redistribution is mediated mainly by vasoconstriction of the splanchnic, renal, cutaneous, and celiac vascular beds and by vasodilatation of the skeletal muscle vasculature (Iriuchijima et al., 1982; Kirby et al., 1987; Kapusta et al., 1989; Zhang et al., 1992, 1996; Anderson and Overton, 1994; Schadt and Hasser, 1998; Blessing, 2003; Mohammed et al., 2014). The increase in skeletal muscle blood flow is primarily due to an increase in the level of circulating catecholamines and activation of β_2_-adrenoceptors in the skeletal muscle beds, whereas vasoconstriction in the visceral vasculature is mediated by catecholamine released from both the adrenal medulla and sympathetic nerve fibers and activation of α-adrenoceptors (Iriuchijima et al., 1982; Knardahl and Hendley, 1990; Zhang et al., 1992, 1996).

An increase in the total peripheral resistance during stress was reported in borderline hypertensive rats (Hatton et al., 1997). However, studies in normotensive animals documented little or no change in this parameter (Hubbard et al., 1986; Martin et al., 1996; Zhang et al., 1996; Schadt and Hasser, 1998), indicating that vasodilation mostly balanced vasoconstriction. Therefore, the increase in cardiac output is possibly the main mechanism mediating the moderate increase in arterial pressure during aversive threats in rodents. In this regard, hemodynamic changes during stress also include an increase in venomotor tone via activation of α_2_-adrenoceptors (Martin et al., 1996; Schadt and Hasser, 1998), which increase venous return and contribute to stress-induced increase in the cardiac output.

#### Cardiac autonomic activity

The tachycardic response during aversive threats is mediated by an increase in the sympathetic tone to the heart (Iwata and LeDoux, 1988; Baudrie et al., 1997; Sgoifo et al., 1999; van den Buuse et al., 2001; Carrive, 2006; Crestani et al., 2010b; Dos Reis et al., 2014). However, stress-induced tachycardic response was enhanced following systemic treatment with muscarinic cholinergic receptor antagonists (Iwata and LeDoux, 1988; Baudrie et al., 1997; Nijsen et al., 1998; Carrive, 2006; Crestani et al., 2009; Dos Reis et al., 2014), indicating an increase in the parasympathetic tone to the heart. Indeed, coactivation of cardiac sympathetic and parasympathetic activities has been documented during aversive situations (Iwata and LeDoux, 1988; Baudrie et al., 1997; Carrive, 2006; Dos Reis et al., 2014). The increase in the parasympathetic tone to the heart allows precise response control, reducing the amplitude of the response, and counteracting excessive cardiac activation associated with cardiac sympathetic system activation alone. The parasympathetic activation and the consequent reduction of cardiac response possibly contributes to a functional state stabilization of the heart (Paton et al., 2005).

#### Modulation of baroreflex function

**Figure 1:**
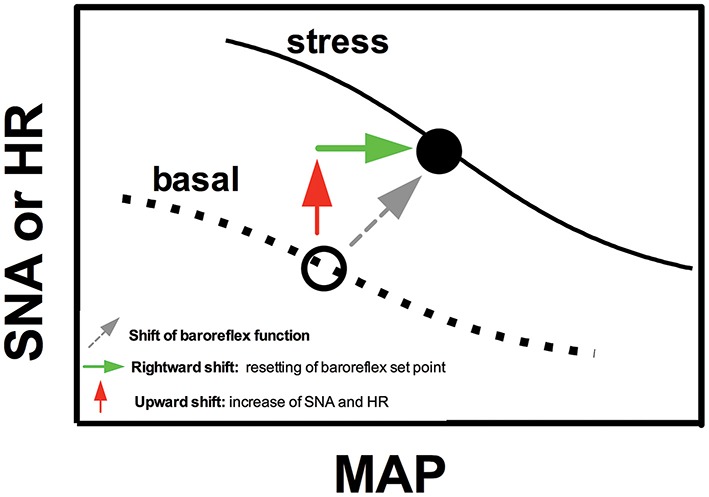
**Schematic representation sketching the modulation of baroreflex function during emotional stress**. Figure schematically represents sigmoid baroreflex curves correlating mean arterial pressure (MAP) and sympathetic nerve activity (SNA) or heart rate (HR) in basal condition (basal) and during an aversive situation (stress). Note that baroreflex curves shifts to the right (green arrow) and upward (red arrow). The rightward shift represents a resetting of the operating point of baroreflex toward higher arterial pressure values, whereas the upward shift is due to increase in SNA and HR during stress (possibly mediated by mechanisms independent of baroreflex resetting). For details, see text in the section “*Cardiovascular responses during acute sessions of stress.*”

Change in baroreflex function is a relevant mechanism controlling cardiovascular and autonomic responses during aversive threats. The baroreflex is an important mechanism for arterial pressure regulation. For example, an increase in arterial pressure activates baroreceptors within the wall of the carotid arteries and aorta that in turn increase cardiac parasympathetic activity and decrease sympathetic activity, leading to decreased HR, vascular resistance, and venous return (Michelini, 1994; Sved and Gordon, 1994). However, as stated above, the increase in arterial pressure during stress is followed by a parallel enhancement in sympathetic activity and HR. Hilton (1963) addressed first the question regarding how parallel increase in blood pressure, HR, and sympathetic activity occurs during aversive situations. Based on the responses obtained by stimulation of the hypothalamic defense area, which mimics the cardiovascular responses induced by stress, baroreflex function was first claimed to be suppressed during defensive behavior (Hilton, 1963; Coote et al., 1979). However, further studies consistently demonstrated that pressor, tachycardic, sympathetic activation, and regional vasoconstrictor responses observed during emotional stress are enhanced in sinoaortic baroreceptor-denervated rats (Norman et al., 1981; Buchholz et al., 1986; Zhang et al., 1996; Dos Reis et al., 2014), indicating an active role of the baroreflex counteracting stress-induced autonomic and cardiovascular responses during defensive responses. Indeed, baroreflex is clearly functional during aversive threats, but it curves for both renal sympathetic nerve activity and HR shifts to the right and upward (Shammas et al., 1988; Hatton et al., 1997; Schadt and Hasser, 1998; Kanbar et al., 2007; Burke and Head, 2009; Crestani et al., 2010b; Miki and Yoshimoto, 2013), allowing a parallel increase in arterial pressure, HR, and sympathetic activity. A schematic representation of the right and upward shift of the baroreflex curve during aversive situations is presented in Figure 1.

The rightward shift represents a resetting of the operating point of the baroreflex toward higher arterial pressure values (Figure 1). Therefore, the baroreflex operates under higher arterial pressure values during stress. This idea is supported by evidence that a decrease in the increase in arterial pressure due to treatment with α-adrenoceptor antagonists (i.e., maintaining arterial pressure values below the new operating point of the baroreflex) enhanced the tachycardic response during aversive situations (Baudrie et al., 1997; Carrive, 2002; Dos Reis et al., 2014), possibly to increase arterial pressure to some preset value. The baroreflex resetting seems to be a mechanism allowing the simultaneous increase in arterial pressure and sympathetic activity/HR rather than generating the cardiovascular responses to stress, once sinoaortic baroreceptor denervation facilitated stress-induced cardiovascular and autonomic adjustments, as stated above (Norman et al., 1981; Buchholz et al., 1986; Zhang et al., 1996; Dos Reis et al., 2014). However, Zhang et al. (1996) reported that vasodilatation in the skeletal muscle vasculature was reduced in sinoaortic baroreceptor-denervated rats, indicating that baroreflex activity resetting may contribute to blood flow redistribution during aversive threats.

The abovementioned evidence indicates that autonomic and cardiovascular responses to stress are mediated by mechanisms acting independent of the changes in baroreflex function. However, the increase in sympathetic activity and HR is responsible for the upward shift of the baroreflex curves (Schadt and Hasser, 1998; Crestani et al., 2010b; Miki and Yoshimoto, 2013; Figure 1). Indeed, the organism operates at higher values of HR and sympathetic activity during stress, inducing a vertical change in the baroreflex curves (Schadt and Hasser, 1998; Crestani et al., 2010b; Miki and Yoshimoto, 2013; Figure 1).

#### Recovery of cardiovascular function after ending stress session

Arterial pressure and HR progressively decrease toward baseline values during recovery from an aversive stimulus, returning to pre-stress levels ~30–60 min after ending stress (McDougall et al., 2000; Vianna and Carrive, 2005; Igosheva et al., 2007; Porter et al., 2007; Crestani et al., 2010b; Krause et al., 2011). Nevertheless, we have previously reported that besides arterial pressure and HR had already returned to pre-stress values 30 min after ending an acute session of restraint stress, the baroreflex activity remained similar to that observed during stress, returning to control values only 60 min after ending the stress session (Crestani et al., 2010b). These findings indicate non-persistent effects of stress on cardiovascular function, but suggest a period of vulnerability during the recovery period, once the baroreflex function is reset to higher arterial pressure values. Nevertheless, persistence of the stress effects on baroreflex activity may be important to counteract excessive activation of depressor mechanisms during the recovery period, thus allowing adequate return of blood pressure to normal values without abrupt falls. Indeed, central and peripheral depressor mechanisms are activated during the recovery period and mediate the return of arterial pressure to resting values (Yip and Krukoff, 2002; D'Angelo et al., 2006).

### Cardiovascular responses to chronic stressors

The physiological and behavioral responses during acute stress sessions constitute important adaptive responses, maintaining homeostasis and ensuring survival (Sterling and Eyer, 1988; Sterling, 2012). However, the frequent/prolonged activation (i.e., over-exposure to stress changes) or generation of inadequate responses (i.e., insufficient responses to individual needs) during stressful events can result in disease development (McEwen and Stellar, 1993; McEwen, 1998; Danese and McEwen, 2012). A situation of over-exposure to stress responses occurs during repeated exposure to aversive threats (i.e., chronic stress; McEwen, 1998). Regarding the cardiovascular consequences of stress, besides reports of acute cardiovascular events such as ventricular arrhythmias, myocardial infarction, and cardiomyopathy (potentially fatal) after exposure to acute stressful situations (Cannon, 1942; Lown et al., 1973; Natelson and Cagin, 1979; Sgoifo et al., 1997, 1999; Ueyama et al., 2002; Mostofsky et al., 2014; Lagraauw et al., 2015), enduring cardiovascular changes (days to years) are mainly provoked by long-term exposure to stressful events (Nalivaiko, 2011). Therefore, the frequency of exposure to stress (chronicity) is an important factor in determining the pattern of stress-induced cardiovascular diseases. Below, we discuss the evidence of enduring changes in arterial pressure, HR, cardiac function, autonomic activity, and baroreflex function that lasts beyond the duration of chronic stress protocols.

#### Arterial pressure

Despite of pronounced neuroendocrine (e.g., increased hypothalamic–pituitary–adrenal, HPA, axis activity), somatic (e.g., reduced body weight gain, adrenal hypertrophy, and thymus atrophy), and behavioral (e.g., anxiety- and depression-like behavior) changes following long-term exposure to stressful events, preclinical studies have failed to consistently reproduce epidemiological findings demonstrating a relationship between chronically stressful life events and hypertension in animals (Timio et al., 1988; Markovitz et al., 2004). Indeed, inconsistent findings have been reported regarding the effects of chronic stress on arterial pressure in animal models. Nalivaiko (2011) reviewed in details publications that investigated the effect of different chronic stress models on arterial pressure in several rat strains. An important consideration raised was the influence of the method used to assess arterial pressure. For example, most studies reviewed by Nalivaiko (2011) that identified a significant hypertensive effect following exposure to chronic stressors used the tail-cuff method (indirect) to measure the arterial pressure. Even in studies that validated the findings obtained through the tail-cuff method by direct methods (through implantation of a catheter into the femoral or carotid artery at the end of the stress protocol), the arterial pressure increase obtained in the direct method was lower than that in the tail-cuff method (Nalivaiko, 2011). Similar analysis of studies published after 2007 (date of the more recent study included in the Nalivaiko's review; Table 1) further reinforces observation regarding the influence of the method used to assess arterial pressure in the identification of stress-induced hypertension.

**Table 1:** **Studies examining the effect of the exposure to chronic stressors in basal parameters of mean arterial pressure (MAP)**.

**Specie/Strain**	**Age/Gender**	**Chronic stressor (length of each session)**	**Protocol stress duration**	**Basal MAP in control**	**MAP change**	**Method of measurement**	**References**
Long-Evans rats	275 g/male	CVS (variable)	15 days	Data not shown	No change (dark and light period)	Telemetry	Goodson et al., 2016
Wistar rat	50 days (adult)/male	CVS (variable)	14 days	103 mmHg	No change	Direct	Almeida et al., 2015
**Wistar rat**	**37 days (adolescent)/male**	**RRS (1 h, 5 days/week)**	**15 weeks**	**118 mmHg (systolic)**	+**30 mmHg**	**TC**	**Bruder-Nascimento et al. 2015**
**Wistar rat**	**28 days (adolescent) 60days (adult) male**	**CVS (variable) RRS (60 min daily)**	**14 days**	**95 mmHg (adult) 97 mmHg (adolescent)**	+**15 mmHg (RRS/adult)** +**10 mmHg (CVS/adult)** +**9 mmHg (CVS/adolescent)**	**Direct**	**Duarte et al., 2015a**
Wistar rat	28 days (adolescent) 60 days (adult) male	CVS (variable) RRS (60 min daily)	10 days	100 mmHg (adult) 97 mmHg (adolescent)	No change	Direct	Duarte et al., 2015b
**Wistar rat**	**Adult**	**RRS (4 h daily)**	**6 weeks**	~**120 mmHg (systolic)**	~+**25 mmHg**	**TC**	**Habib et al., 2015**
**WKY and SHR rats**	**5 weeks/male and female**	**Crowding (continuous)**	**2 weeks**	~**160 mmHg (SHR)** ~**110 mmHg (WKY) (systolic)**	+**20 mmHg (only SHR male)**	**TC**	**Ledvenyiova-Farkasova et al., 2015**
Wistar rat	350 g (adult)/male	CVS (10–14h, 7 days/week)	8 weeks	104 mmHg	No change	TC	Matchkov et al., 2015
Wistar rat	300–400 g (adult)/male	CVS (variable, 7 days/week)	8 weeks	95 mmHg	No change	Direct	Demirtas et al., 2014
Wistar rat	200–250 g (adult)/male	CVS (variable, 7 days/week)	8 weeks	99 mmHg (systolic)	No change	TC	Ismail et al., 2014
WKY, BHR, and SHR rats	5 weeks/female	Crowding	2 weeks	100 mmHg (WKY) 110 mmHg (BHR) 160 mmHg (SHR) (systolic)	No change	TC	Slezak et al., 2014
BALB/cJ mice	9 weeks/male-female	CVS (variable, 7 days/week)	8 weeks	Male 87 mmHg Female 91 mmHg	No change	Direct	Stanley et al., 2014
**Sprague–Dawley rat**	**8 weeks/male**	**RRS (2 h daily)**	**14 days**	**Data not shown**	**Increase**	**TC**	**Yang et al., 2014**
Wistar rat	250–300 g/male	CVS (variable, 7 days/week)	8 weeks	102 mmHg (systolic)	No change	TC	Bayramgurler et al., 2013
Wistar rat	7–10 weeks/male	Footshock (40 min, 3−7 days/week)	6 weeks	~110 mmHg	No change (*increased SAP during, but not after stress*)	Telemetry	Bobrovskaya et al., 2013
WKY rat	12 weeks/male	Crowding	8 and 12 weeks	~110 mmHg (8 weeks) ~105 mmHg (12 weeks) (systolic)	No change	TC	Puzserova et al., 2013
Sprague–Dawley rat	250–300 g/male	Social defeat	4 days + 5 days	102 mmHg	No change	Direct	Sévoz-Couche et al., 2013
Wistar rat	5–6 weeks/male	CVS (12 h, 7 days/week)	4 or 8 weeks	121 mmHg	No change	TC	Bouzinova et al., 2012
**Wistar rat**	**30 days (adolescent)/male**	**RRS (1 h, 5days/week)**	**15 weeks**	~**120 mmHg (systolic)**	~+**30 mmHg**	**TC**	**Bruder-Nascimento et al., 2012**
**Sprague–Dawley rat**	**275–300 g/male**	**RRS (60 min daily)**	**5 days**	~**100 mmHg**	+**10 mmHg** (light period)	**Telemetry**	**Daubert et al., 2012**
**Rabbits**	**2 kg(adult)/male**	**CVS**+**HD**	**8 and 16 weeks**	**116 mmHg (8 week) 118 mmHg (16 week) (systolic)**	+**29 mmHg (8 week)** +**46 mmHg (16 week)**	**Indirect**	**Lu et al., 2012**
WKY rat	12 weeks/male	Crowding	8 weeks	~110 mmHg (systolic)	No change	TC	Puzserova et al., 2012
Wistar rat	5–6 weeks/male	Social isolation	14 days	~90 mmHg (diastolic) ~125 mmHg (systolic)	No change	Telemetry	Tsvirkun et al., 2012
Wistar rat	230–250 g/male	CVS (variable, 7 days/week)	2 weeks	~120 mmHg (systolic)	No change	Direct	Xie et al., 2012^*^
Sprague–Dawley rat	250–275 g (adult)/male	CVS (variable, 7 days/week)	2 weeks	~105 mmHg	No change (24 h recording)	Telemetry	Flak et al., 2011
**WKY and SHR rats**	**Adult/male**	**Crowding**	**8 weeks**	**110 mmHg (WKY) 189 mmHg (SHR)**	+**8 mmHg (only SHR)**	**TC**	**Ravingerova et al., 2011**
**WKY and SHR/y rat**		**Colony social stress**	**6 months**	**120 mmHg (WKY) 140 mmHg (SHR/y) (systolic)**	+**20 mmHg (WKY)** +**15 mmHg (SHR)**		**Toot et al., 2011**
**BHR and Wistar rat**	**15 weeks (adult)/male and female**	**Crowding**	**6 weeks**	~**110 mmHg (Wistar)** ~**130 mmHg/male** ~**120 mmHg/female (BHR)**	+**10 mmHg (only BHR male)**	**TC**	**Bernatova et al., 2010**
Sprague–Dawley rat	8–10 weeks (adult)/male	CVS (variable, 7 days/week)	4 weeks	~115 mmHg	No change	Direct	Cudnoch-Jedrzejewska et al., 2010
WKY	12 weeks (adult)/male	Crowding	8 weeks	111 mmHg (systolic)	No change	TC	Puzserova and Bernatova, 2010
BHR/WKY rats	BHR-385 g/WKY-414 g (adults)	RRS (1–2 h, 5 days/week)	21–25 days	BHR-122 mmHg WKY-110 mmHg	No change	Direct	Bechtold et al., 2009
**Sprague–Dawley rat**	**250–300 g/male**	**CVS (variable)**	**4 weeks**	~**120 mmHg**	~+**6 mmHg**	**Direct (24 h recovery)**	**Grippo et al., 2008**
WKY rat	12 weeks/male	Crowding	8 weeks	110 mmHg (systolic)	No change	TC	Bernatova et al., 2007
**Wistar, BHR, and SHR**	**12 weeks/male**	**Crowding**	**8 weeks**	**Wistar 111 mmHg BHR 135 mmHg SHR 182 mmHg (systolic)**	**No change** +**6 mmHg** +**9 mmHg**	**TC**	**Bernatowa et al., 2007**

BHR, borderline hypertensive rat; CVS, chronic variable stress; Dahl-S, Dahl salt-sensitive rats; Dahl-R, Dahl salt-resistant rats; HD, high-cholesterol diet; ISIAH-inherited stress-induced arterial hypertension; LE, Long Evans rat; RRS, repeated restraint stress; SHR, spontaneously hypertensive rats; SHR/Y, borderline hypertensive consomic rat strain; WKY, Wistar-Kyoto rat.

Bold indicates studies that identified effect of chronic emotional stress in baseline MAP.

* Cardiovascular recording in anesthetized animals.

Animals need to be restrained (similar to the restraint stress model) in the tail-cuff method, and marked pressor and tachycardic responses are observed, even following recommended habituation (Grundt et al., 2009). Based on this, Nalivaiko (2011) proposed that the identification of hypertensive effect preferentially by the tail-cuff method would be due to a more vigorous reaction of the animals stressed to the tail-cuff procedure. Hence, it has been reported that exposure to chronic stressors increases pressor response during acute aversive stimuli (Grippo et al., 2002, 2006; Cudnoch-Jedrzejewska et al., 2010, 2014; Maslova et al., 2010).

Another factor mentioned by Nalivaiko (2011) could be the possible influence of coping strategies in models providing actual or perceived control over a stressful situation, which may occur, for example, in highly predictable protocols (e.g., during repeated exposure to the same stressor). We recently addressed this issue by comparing the impact on arterial pressure (measured by the direct method) of a predictable (repeated restraint stress, RRS) vs. an unpredictable (chronic variable stress, CVS) chronic stress protocol (Duarte et al., 2015a) (see the next section for details). We observed a similar small pressor effect (+10–15 mm Hg) following exposure to either RRS or CVS for 10 days (Duarte et al., 2015a). Other studies using longer CVS protocols (4–8weeks) reported either similar effect (~+8 mm Hg) (Grippo et al., 2008) or absence of changes on arterial pressure measured by either direct methods or telemetry (Grippo et al., 2002, 2006; Cudnoch-Jedrzejewska et al., 2010; Demirtas et al., 2014; Stanley et al., 2014). In addition, Bobrovskaya et al. (2013) did not identify changes in mean arterial pressure measured by telemetry (only a small increase in systolic arterial pressure was identified) following repeated exposure to footshock (more intense aversive stimulus) for 6 weeks in an unpredictable schedule. Thus, the small hypertensive effect obtained by direct measurement of arterial pressure seems to be independent of intensity, predictability, and duration of chronic stress protocol.

An additional factor considered in our review (Table 1) was an elevated baseline arterial pressure in the control group, which would indicate some degree of stress induced by the experimental procedure (independent of chronic stressor) that in turn could buffer effects of chronic stress. However, independently of the chronic stress paradigm, a similar small hypertensive effect (~10 mm Hg) was observed following long-term stress exposure in studies wherein baseline mean arterial pressure in control groups ranged from 95 to 120 mmHg (Grippo et al., 2008; Daubert et al., 2012; Duarte et al., 2015a; Cruz et al., 2016) (Table 1). It has also been documented that cardiovascular dysfunctions induced by chronic stressors may be related to animals age (Crestani, 2016); hence, an influence of age (e.g., young vs. adult) was considered in this review (Table 1). However, the influence of age seems to be related to the type of chronic stressor. For example, social isolation for 3 weeks increased arterial pressure in adolescent rats, without affecting this parameter in adult animals (Cruz et al., 2016). In contrast, RRS induced a mild hypertensive effect in adult animals alone (Duarte et al., 2015a).

#### Heart rate and cardiac dysfunctions

More consistent results have been reported for HR. Table 2 summarizes the main findings of changes in baseline HR following exposure to chronic stressors. Increase in baseline HR has been reported in many studies, but this effect seems to be related to the chronic stressor type. Indeed, resting tachycardia has been consistently documented following exposure to two animal models of chronic stress: the CVS and chronic social isolation in prairie voles (a highly social rodent species; Table 2). Resting tachycardia induced by both these models was independent of the protocol length. For example, increased baseline HR was observed following exposure to protocols ranging from 10 days to 8 weeks of CVS (Grippo et al., 2002, 2008; Mercanoglu et al., 2008; Bouzinova et al., 2012; Duarte et al., 2015a) and 5 days to 8 weeks of social isolation in prairie voles (Grippo et al., 2007, 2011; McNeal et al., 2014). Moreover, the increase in HR was not affected by baseline HR in control animals (indicative of some degree of stress of the experimental procedure, see discussion above) and by animal age (Table 2).

**Table 2:** **Studies examining the effect of the exposure to chronic stressors in basal parameters of heart rate (HR)**.

**Specie/Strain**	**Age/Gender**	**Chronic stressor (length of sessions)**	**Protocol length**	**HR basal in control group**	**HR change**	**Method of measurement**	**References**
Long-Evans rats	275 g/male	CVS (variable)	15 days	Data not shown	No change (dark and light period)	Telemetry	Goodson et al., 2016
Wistar rat	50 days (adult)/male	CVS (variable)	14 days	368 bpm	No change	Direct	Almeida et al., 2015
WKY rat	3 months male	Social defeat (30 min contact without physical interaction/10 min physical interaction)	5 weeks	~320 bpm	No change		**Carnevali et al., 2015**
Wistar rat	28 days (adolescent) 60days (adult) male	Social isolation (continuous)	3 weeks	338 bpm (adult) 371 bpm (adolescent)	No change	Direct	Cruz et al., 2016
**Wistar rat**	**28 days (adolescent) 60 days (adult) male**	**CVS (variable)** RRS (60 min daily)	**14 days**	**343 bpm (adult) 390 bpm (adolescent)**	+**30 bpm (CVS/adolescent)** +**32 mmHg (CVS/adult)** RRS-No change	**Direct**	**Duarte et al., 2015a**
**Wistar rat**	**28 days (adolescent) 60days (adult) male**	**CVS (variable)** RRS (60 min daily)	**10 days**	**329 bpm (adult) 387 bpm (adolescent)**	+**34 bpm (CVS/adolescent)** +**11 mmHg (CVS/adult)** RRS-No change	**Direct**	**Duarte et al., 2015b**
WKY and SHR rats	5 weeks/male and female	Crowding (continuous)	2 weeks	Data not shown	No change	TC	Ledvenyiova-Farkasova et al., 2015
**Wistar rat**	**350 g(adult)/male**	**CVS (10–14 h)**	**8 weeks**	~**350 bpm**	+**100 bpm**	**TC**	**Matchkov et al., 2015**
Wistar rat	200–250 g/male	Footshock (1 h daily)	7 days	~300 bpm	No change	Isolated heart	Rakhshan et al., 2015
Wistar rat	200–250 g (adult)/male	CVS	8 weeks	344 bpm	No change	TC	Ismail et al., 2014
**Prairie voles**	**60–90 days/male**	**Social isolation**	**5 days**	~**400 bpm**	~+**50 bpm**	**Telemetry**	**McNeal et al., 2014**
WKY, BHR, and SHR rats	5 weeks/female	Crowding	2 weeks	420 bpm (WKY) 440 bpm (BHR) 497 bpm(SHR)	No change	TC	Slezak et al., 2014
Wistar rat	7–10 weeks/male	Footshock (40 min, 3–7 days/week)	6 weeks	~400 bpm	No change	Telemetry	Bobrovskaya et al., 2013
WT Groningen rat	10 weeks/male	Social defeat (12 episodes/75min)	25 days + 2days	~167ms (R-R interval)	No change	Telemetry	Carnevali et al., 2013b
**Sprague–Dawley rat**	**250–300 g/male**	**Social defeat**	**4 days** + **5 days**	**321 bpm**	+**24 bpm**	**Electrocardiogram**	**Sévoz-Couche et al., 2013**
**Wistar rat**	**5–6 weeks/male**	**CVS (12 h)**	**4 or 8 weeks**	**354 bpm**	+**90 bpm**	**TC**	Bouzinova et al., 2012
5-HT1A KO and WT mice	12 weeks/male	Social defeat (cohabited with resident, 5 defeat episodes)	15 days + 3 days	~530 bpm	No change	Telemetry	Carnevali et al., 2012
Sprague–Dawley rat	275–300 g/male	RRS (60 min daily)	5 days	~375 bpm	No change (day time and night time)	Telemetry	Daubert et al., 2012
**Prairie voles**	**81 days/female**	**Social isolation**	**4 weeks**	~**390 bpm**	+**20 bpm**	**Telemetry**	Grippo et al., 2012a
**Prairie voles**	**60–90 days/female**	**Social isolation**	**4 weeks**	~**350 bpm**	~+**70 bpm**	**Telemetry**	**Grippo et al., 2012b**
**Rabbits**	**2 kg(adult)/male**	**CVS**+**HD**	**8 and 16 weeks**	**257 bpm (8 week) 254 bpm (16 week) (systolic)**	+**25 bpm (8 week)** +**91 bpm (16 week)**	**Indirect**	**Lu et al., 2012**
WKY rat	12 weeks/male	Crowding	8 weeks	~400 bpm	No change	TC	Puzserova et al., 2012
Wistar rat	5–6 weeks old	Social isolation (standard plastic cages)	14 days	~300 bpm	No change	Telemetry	Tsvirkun et al., 2012
Wistar rat	230–250 g/male	CVS (variable, 7 days/week)	2 weeks	~390 bpm	No change	Direct	Xie et al., 2012^*^
Sprague–Dawley rat	275–300 g/male	Social defeat 30 min/session	7 days	370 bpm	No change	Telemetry	Wood et al., 2012
**Sprague–Dawley rat**	**10 weeks/male**	**Footshock (1 h daily)**	**5 days**	**416 bpm (night time) 359 bpm(day time)**	**–23 bpm (night time) –20 bpm (day time)**	**Telemetry**	**Carnevali et al., 2011**
Sprague–Dawley rat	250–275 g (adult)/male	CVS (variable, 7 days/week)	2 weeks	~350 bpm	No change (24 h recording)	Telemetry	Flak et al., 2011
**Prairie voles**	**75 days/female**	**Social isolation**	**2 and 4 weeks**	~**390 bpm**	~+**35 bpm(24 h recording)**	**Telemetry**	**Grippo et al., 2011**
WKY and SHR rats	Adult/male	Crowding	8 weeks	306 bpm (WKY) 317 bpm(SHR)	No change	Isolated heart	Ravingerova et al., 2011
Sprague–Dawley rat	8–10 weeks (adult)	CVS	4 weeks	~350 bpm	No change	Direct (24 h recovery)	Cudnoch-Jedrzejewska et al., 2010
**Prairie voles**	**60–90 days/female**	**Social isolation**	**2 and 4 weeks**	~**360 bpm**	~+**40 bpm(24 h recording)**	**Telemetry**	**Grippo et al., 2010**
WKY	12 weeks (adult)/male	Crowding	8 weeks	385 bpm	No change	TC	Puzserova and Bernatova, 2010
BHR/WKY rats	BHR-385 g/WKY-414 g (adults)	RRS (1–2 h, variable)	21–25 days	BHR-295 bpm WKY-312bpm	No change	Direct (24 h recovery)	Bechtold et al., 2009
**Prairie voles**	**60–90 days/female**	**Social isolation**	**2 and 4 weeks**	~**360 bpm**	~+**40 bpm**	**Telemetry**	**Grippo et al., 2009**
**Sprague–Dawley rat**	**250–300 g/male**	**CVS (variable)**	**4 weeks**	~**380 bpm**	~+**20 mmHg**	**Direct (24 h recovery)**	**Grippo et al., 2008**
**Sprague–Dawley rat**	**250–300 g/male**	**CVS (variable)**	**20 days**	**253 bpm**	+**24 bpm**	**ECG**	Mercanoglu et al., 2008^*^
Wistar, BHR, and SHR	12 weeks/male	Crowding	8 weeks	Wistar411 bpm BHR415 bpm SHR453 bpm	No change	TC	Bernatowa et al., 2007
WKY rat	12 weeks/male	Crowding	8 weeks	335 bpm	No change	TC	Bernatova et al., 2007
**Prairie voles**	**60–90 days/female**	**Social isolation**	**2 and 4 weeks**	~**360 bpm**	~+**40 bpm**	**Telemetry**	**Grippo et al., 2007**
BHR	15 weeks/male	Crowding	6 weeks	446 bpm	No change	TC	Bernatova and Csizmadiova, 2006
**Sprague–Dawley rat**	**250–300 g/male**	**CVS(variable, 7 days/week)**	**4 weeks**	**371 bpm**	+**26 bpm**	**Direct (48 h recovery)**	**Grippo et al., 2006**
**Sprague–Dawley rat**	**300–400 g/male**	**CVS(variable)**	**4 weeks**	~**240 bpm**	~+**20 bpm**	**Direct(24 h recovery)**	Grippo et al., 2004^*^
Swiss CD-1 mice	12 weeks, male	Social defeat (cohabited with resident, 5 min agonistic interaction/day)	15 days	~530 bpm (day) ~600 bpm (night)	No change (*post-stress period*)	Telemetry	Costoli et al., 2004
Sprague–Dawley rat	5 weeks/male	Restraint/heat stress (45 min, twice daily)	2 weeks	447 bpm	No change	Direct	Porter et al., 2004
Swiss CD-1 mice	12 weeks, male	Social defeat (cohabited with resident, 5-15min agonistic interaction/day)	15 days	~500 bpm (day) ~600 bpm (night)	No change (*post-stress period*)	Telemetry	Bartolomucci et al., 2003
**Sprague–Dawley rats**	**250–300 g (adult)/male**	**CVS**	**4 weeks**	**329 bpm**	+**32 bpm**	**Direct (48 h recovery)**	**Grippo et al., 2003**
**Sprague–Dawley rat**	**300-400 g (adult)/male**	**CVS**	**4 weeks**	**364 bpm**	+**18 bpm**	**Direct (72 h recovery)**	**Grippo et al., 2002**
WKY	12 weeks/male	RRS(10–20 min daily)	5 days	340 bpm	No change	Direct(24 h recovery)	Conti et al., 2001
WT Groningen rat	20 weeks/male	Social defeat/victory (15 min/day)	10 days	200 ms (R-R interval)	No change (*basal*)	Telemetry	Sgoifo et al., 2001
WKY and SHR	15–16 weeks/male	RRS (60 min dalily, isolated)	10 days	WKY 285 bpm	No change	Telemetry	McDougall et al., 2000
				SHR 288 bpm	No change		
Lister hooded rat	180 g/male	Social defeat (10 min/day)	10 days	440 bpm	No change	Telemetry	Chung et al., 1999
**Wistar rat**	**150–250 g/male**	**Social isolation RRSCrowding**	**7, 15, and 30 days**	**362 bpm**	+**25 bpm (isolation, 7 days)** +**68 bpm (RRS, 7 days)**	**Electrocardiogram**	**Nagaraja and Jeganathan, 1999**
Sprague–Dawley rat	–	RRS (1, 1.5, or 2 h daily)	8–14 days	430 bpm	No change	Direct (30 min after surgery)	Scheuer and Mifflin, 1998
LE rat	280–340 g/male	Social defeat	5 days	~340 bpm	No change (24 h recording) *decreased circadian rhythmicity*	Telemetry	Tornatzky and Miczek, 1993
Sprague–Dawley rat	275–300 g/male	RRS Cold swim Footshock (30 min/daily)	26 days	350 bpm	No change	Direct (24 h recovery)	Konarska et al., 1989
				338 bpm	No change		
				346 bpm	No change		
Dahl-S	70–80 days (adult)	Footshock (25 min)	5 days	440 bpm	No change	TC	Adams et al., 1987
		Social Defeat (25 min)	5 days	440 bpm	No change		
Dahl-S+salt	70–80 days (adult)	Social Defeat (25 min)	6 days	452 bpm	No change	TC	Adams and Blizard, 1986
Dahl-R+salt				388 bpm			
Brattleboro and LE rats	Brattleboro 280–340 g LE 310–380 male	Social isolation	5 and 11 days	~375 bpm (LE) ~410 bpm (Brattleboro)	No change	TC	Gardiner and Bennett, 1983

BHR, borderline hypertensive rat; CVS, chronic variable stress; Dahl-S, Dahl salt-sensitive rats; Dahl-R, Dahl salt-resistant rats; ECG, electrocardiogram; HD, high-cholesterol diet; ISIAH, inherited stress-induced arterial hypertension; KO, knockout mice; LE, Long Evans rat; RRS, repeated restraint stress; SHR, spontaneously hypertensive rats; WKY, Wistar-Kyoto rat.

Bold indicates studies that identified effect of chronic stress in baseline HR.

* Cardiovascular recording in anesthetized animals.

Although, most studies investigating the effect of chronic social isolation in prairie voles have used female animals (Table 2), resting tachycardia was also reported in male prairie voles following a period of social isolation (McNeal et al., 2014). CVS-induced increase in HR was evaluated only in males animals, but was documented in both Wistar and Sprague–Dawley rats (Grippo et al., 2002, 2006, 2008; Bouzinova et al., 2012; Duarte et al., 2015a; Matchkov et al., 2015), indicating that the effect is independent of the rat strain. As discussed for the effect of stress on arterial pressure, we note higher responses in studies that measured HR by the tail-cuff method compared with data obtained by other methods (Table 2). As discussed above, it can be related to a more vigorous reaction of the stressed animals to the tail-cuff procedure (Nalivaiko, 2011).

Despite the absence of changes in baseline HR, some models induce profound changes in cardiac function. For example, repeated social defeat episodes caused accumulation of fibrous tissue in the left ventricular myocardium, maladaptive cardiac hypertrophy, changes in electrical conduction system of the heart (e.g., reduced myocardial refractoriness and impaired conduction), and increased susceptibility to cardiac arrhythmias (Gelsema et al., 1994; Costoli et al., 2004; Carnevali et al., 2013b, 2015; Sgoifo et al., 2014). Disruption of the circadian rhythm for HR has also been reported following repeated exposure to social defeat (Tornatzky and Miczek, 1993; Sgoifo et al., 2002; Carnevali et al., 2015).

RRS is another chronic stressor that does not induce significant changes in baseline HR (Table 2), but cardiac hypertrophy (Nagaraja and Jeganathan, 1999; Bruder-Nascimento et al., 2012; Duarte et al., 2015a), electrocardiogram (ECG) abnormality, myocardium injury, and cardiac dysfunction (Zhao et al., 2007; Roth et al., 2015) have been reported in animals exposed to this experimental model of stress. Moreover, RRS increased the size of infarction and the incidence of potentially fatal arrhythmias induced by myocardial ischemia (Scheuer and Mifflin, 1998), increased plaque formation in the coronary arteries and occurrence of myocardial infarctions in mice susceptible to atherosclerosis (Roth et al., 2015), and exacerbated hypertension and left ventricular hypertrophy, fibrosis, and diastolic dysfunction related to metabolic syndrome (Habib et al., 2015; Matsuura et al., 2015). These results indicate that RRS may affect the outcome of cardiac diseases.

Consistent findings have also indicated that crowding stress (i.e., social stress that causes competition for resources such as space, food, and water) does not affect baseline HR (Table 2). Nevertheless, analysis of responses to myocardial ischemia indicated an impairment of post-ischemic recovery of cardiac mechanical function and aggravation of tachyarrhythmia and ventricular fibrillation in animals subjected to crowding stress (Ravingerova et al., 2011; Ledvenyiova-Farkasova et al., 2015), suggesting that exposure to this social stressor reduces the tolerance to cardiac ischemia. Maladaptive cardiac hypertrophy, ECG abnormality, and impairment of coronary perfusion of the myocardium have also been reported following exposure to crowding stress (Nagaraja and Jeganathan, 1999; Ravingerova et al., 2011).

In addition to causing tachycardia, cardiac dysfunctions have also been reported following exposure to either CVS or chronic social isolation in prairie voles. Indeed, increased susceptibility to experimentally and stress-induced cardiac arrhythmias was reported in animals subjected to either stressor (Grippo et al., 2004, 2010, 2012a; Liang et al., 2015), and CVS increased the infarcted area after myocardial ischemia (Mercanoglu et al., 2008). These findings indicate that these experimental models of stress may increase the vulnerability and severity of cardiac complications. In addition, cardiac contractile dysfunction was reported in animals subjected to CVS protocols (Grippo et al., 2006; Xie et al., 2012).

Studies in the literature also investigated a possible impact of chronic stressors in the pacemaker activity of the sinoatrial node, referred as intrinsic HR. It can be assessed experimentally by dual blockade of sympathetic and parasympathetic activities of the heart via combined systemic treatment with β-adrenoceptor antagonists (i.e., sympathetic blocker; e.g., propranolol or atenolol) and muscarinic cholinergic receptors (i.e., parasympathetic blocker; e.g., methylatropine). However, intrinsic HR was not affected after exposure to either CVS (Grippo et al., 2002; Almeida et al., 2015; Duarte et al., 2015a), chronic social isolation in prairie voles (Grippo et al., 2007, 2009; McNeal et al., 2014), or RRS (Duarte et al., 2015a). This finding seems to be independent of the duration of chronic stress protocol. For example, intrinsic HR was investigated in CVS protocols ranged from 10 days to 4 weeks (Grippo et al., 2002; Almeida et al., 2015; Duarte et al., 2015a), whereas social isolation in prairie voles ranged from 5 days to 4 weeks (Grippo et al., 2007; McNeal et al., 2014). To the best of my knowledge, a possible impact of other animal models in cardiac pacemaker activity has never been evaluated.

#### Autonomic activity

Reduction in HR variability following exposure to different animal models of chronic stress has been described (Grippo et al., 2002, 2009; Wood et al., 2012; Sévoz-Couche et al., 2013), which indicates changes in cardiac autonomic activity. Indeed, analysis of cardiac autonomic activity by either power spectral analysis of oscillatory components of HR or pharmacological blockade of cardiac sympathetic (e.g., treatment with propranolol) and parasympathetic (e.g., treatment with methylatropine) activities indicated significant stress-induced changes in cardiac autonomic activity. Nevertheless, these alterations seem to be specific to the chronic stressor type. For example, pharmacological blockade of cardiac autonomic activity revealed that CVS increased the sympathetic tone to the heart, without significantly affecting cardiac parasympathetic activity (Grippo et al., 2002; Duarte et al., 2015a). It was also evidenced by demonstration of an increase in LF/HF ratio of HR variability (Bundzikova-Osacka et al., 2015), which indicates a change in cardiac sympathovagal balance toward a sympathetic predominance. In contrast, the changes in cardiac autonomic activity induced by chronic social isolation in prairie voles, evidenced by pharmacological autonomic blockade, were characterized by both a decrease in parasympathetic and an increase in sympathetic tone to the heart (Grippo et al., 2007, 2009; McNeal et al., 2014). Despite specific changes in sympathetic and parasympathetic activities, the changes induced by either CVS or social isolation in prairie voles were characterized by a change in cardiac sympathovagal balance toward a sympathetic predominance, which is in line with the resting tachycardia induced by these experimental models. Moreover, the increase in sympathetic contribution of cardiac autonomic balance corroborates the increased susceptibility to cardiac arrhythmias induced by both models (Grippo et al., 2004, 2006, 2012a), as well as with cardiac contractile dysfunction (Grippo et al., 2006; Xie et al., 2012) and increased severity of myocardial ischemia (Mercanoglu et al., 2008) observed after exposure to CVS. Studies have not reported changes in cardiac sympathovagal balance following exposure to RRS protocols (Daubert et al., 2012; Duarte et al., 2015a,b), which is in line with the absence of changes in baseline HR.

Cardiac autonomic imbalance has been reported in Sprague–Dawley rats subjected to repeated sessions of social defeat (Wood et al., 2012; Sévoz-Couche et al., 2013). By analyzing HR variability, results indicated a shift in sympathovagal balance toward sympathetic predominance (Wood et al., 2012; Sévoz-Couche et al., 2013), which was likely mediated by both a decrease in parasympathetic activity and increase in sympathetic tone to the heart (Sévoz-Couche et al., 2013). This finding is in line with the increase in baseline HR observed in Sprague–Dawley rats following repeated exposure to social defeat (Sévoz-Couche et al., 2013). However, Sprague–Dawley rats seem to be selectively susceptible to resting tachycardia induced by repeated social defeat once studies did not identify changes in baseline HR in either mice (Bartolomucci et al., 2003; Costoli et al., 2004; Carnevali et al., 2012) or wide-type Groningen (Sgoifo et al., 2001; Carnevali et al., 2013b), Long-Evans (Tornatzky and Miczek, 1993), Wistar-Kyoto (Carnevali et al., 2015), Lister hooded (Chung et al., 1999), and Dahl-s (Adams and Blizard, 1986; Adams et al., 1987) rats. Nevertheless, differences in experimental protocol may also account for the differences in findings of autonomic/cardiovascular changes because exposures to social defeat in different studies ranged from 5 to 25 days (Table 2). Moreover, the defeated animal cohabited with its aggressor in some protocols, being subjected to intermittent episodes of aggressive interaction but with continuous sensorial contact (Bartolomucci et al., 2003; Costoli et al., 2004), whereas animals were exposed only to intermittent episodes of social defeat in other studies (Sgoifo et al., 2001; Carnevali et al., 2013b), including those that identified changes in HR (Wood et al., 2012; Sévoz-Couche et al., 2013).

The increase in sympathetic activity induced by chronic stressors seems not to be restricted to the heart. For instance, Grippo et al. (2008) reported that a 4-week CVS protocol increased lumbar sympathetic nerve activity. In addition, an increase in tyrosine hydroxylase expression and activity has been reported in the sympathetic ganglia and adrenal medulla following exposure to chronic stressors (Nankova et al., 1994, 1996; Bobrovskaya et al., 2013). The relevance of the response in the adrenal medulla is unclear since changes in plasma concentration of adrenaline and noradrenaline were not identified following exposure to several chronic stress protocols, including RRS, footshock, social isolation, crowding, and CVS (Konarska et al., 1989; Dronjak et al., 2004; Gavrilovic et al., 2005; Spasojevic et al., 2009). Therefore, sympathoexcitation induced by chronic stressors seems to be mediated by an increase in activity of sympathetic nerves rather than changes in the sympatho-adrenomedullary system.

Long-term potentiation was reported in the sympathetic ganglia of animals subjected to cage-switch stress for 4 weeks (Alkadhi et al., 2005). It is an activity-dependent sustained increase in ganglionic transmission, which is similar to that described in the brain. Therefore, this sustained enhancement of synaptic efficacy may constitute an important mechanism underlying the increase in sympathetic tone following long-term exposure to aversive stimuli (Alkadhi et al., 2005).

#### Baroreflex function

Changes in the baroreflex control of HR were reported following exposure to several chronic stressors, including CVS, RRS, repeated social defeat stress, and chronic social isolation (Conti et al., 2001; Porter et al., 2004; Daubert et al., 2012; Xie et al., 2012; Sévoz-Couche et al., 2013; Almeida et al., 2015; Duarte et al., 2015a; Cruz et al., 2016). However, the effects in the baroreflex activity seem to be dependent on the duration of chronic stress. For example, exposure to CVS protocols of either 10 (Duarte et al., 2015a) or 14 days (Xie et al., 2012; Almeida et al., 2015) induced changes in the baroreflex control of HR, but a 4-week protocol did not affect baroreflex HR responses (Grippo et al., 2008). Nevertheless, the reflex increase in lumbar sympathetic nerve activity induced by hypotension was decreased in animals following exposure to a CVS protocol for 4 weeks (Grippo et al., 2008), indicating that lumbar sympathetic nerve and cardiac autonomic innervation are affected differently by CVS. In addition, different effects of CVS in reflex bradycardia induced by increased blood pressure were obtained when baroreflex function was evaluated in animals anesthetized (facilitation; Xie et al., 2012) and unanesthetized rats (impairment; Almeida et al., 2015; Duarte et al., 2015a), indicating that anesthesia may affect the analysis of cardiovascular changes induced by chronic stressors.

Most studies describing stress effects evaluated the baroreflex activity using the classical pharmacological approach (i.e., arterial pressure changes were induced by intravenous infusion of vasoactive agents). However, analysis of the baroreflex responses over the physiological range of fluctuations in arterial pressure without any pharmacological manipulations (i.e., spontaneous baroreflex) has also provided evidence of an impact of chronic stressors, but the effects are stress-specific type. For example, changes in spontaneous baroreflex activity were reported in animals subjected to repeated exposure to social defeat (Sévoz-Couche et al., 2013) and CVS (Almeida et al., 2015), but not following RRS (Daubert et al., 2012; Duarte et al., 2015b). Therefore, an impact of RRS in baroreflex activity was evidenced by the classical pharmacological approach alone (Conti et al., 2001; Porter et al., 2004; Duarte et al., 2015a). Acute ablation of specific central nervous system regions has different effects on the baroreflex responses assessed by the classical pharmacological approach and the sequence analysis technique (Crestani et al., 2010a; de Andrade et al., 2014), indicating that differences in the neural circuitry of reflex responses within the narrow range of physiological variations and during more pronounced arterial pressure changes could explain the specific influence of RRS on the baroreflex responses over the full range of arterial pressure changes. Nevertheless, further studies are necessary to clarify this issue.

The studies that evaluated cardiovascular function in unanesthetized animals provided evidence that both CVS (Grippo et al., 2008; Almeida et al., 2015; Duarte et al., 2015a) and repeated social defeat stress (Sévoz-Couche et al., 2013) impaired the baroreflex function. Impairment of the baroreflex function is proposed to be involved in the physiopathology of hypertension (Grassi et al., 2006; Honzikova and Fiser, 2009), and is associated with overactivity of sympathetic activity (Grassi et al., 2004). Therefore, sympathoexcitation and mild hypertension (reported in some studies, Table 1) induced by these chronic stressors may be mediated by changes in the baroreflex activity. In contrast, an increase in baroreflex sensitivity was observed following exposure to RRS (Conti et al., 2001; Duarte et al., 2015a). Moreover, a recent study did not identify an impact of chronic social isolation in baroreflex function in adult rats (Cruz et al., 2016). Therefore, an involvement of baroreflex changes in the physiopathology of stress-induced cardiovascular complications is dependent on the chronic stress type.

## Cardiovascular responses to stress: influence of predictability

Most studies that characterized the influence of predictability of stressor in its responses evaluated alterations in neuroendocrine function, behavioral responses, somatic parameters, and brain morphology/function. Indeed, a possible difference in the impact of predictable vs. unpredictable stressors in cardiovascular function and autonomic activity was addressed only recently (Duarte et al., 2015a,b). Therefore, before discussing the influence of predictability in cardiovascular/autonomic responses to stress, a summary of the impact of predictable vs. unpredictable stressors in the HPA axis, anxiety- and depression-like behaviors, and somatic parameters is presented in order to discuss experimental results in rodents that characterized the influence of predictability of stressor stimulus and its consequences.

### Characterization of influence of predictability

The impact of predictability of stressor stimulus was initially examined by comparing responses to signaled (e.g., aversive stimulus preceded by light) vs. unsignaled footshock. Some of these studies indicated that signaling minimized stress reactions (e.g., corticosterone release) and stress-induced pathology (e.g., stomach ulceration; Perkins, 1955; Seligman, 1968; Weiss, 1970), which supported the hypothesis that a reliable predictor of an aversive stimulus minimizes its responses (Seligman, 1968). However, these results were not consistent (Brady et al., 1962; Paré, 1971; Bassett et al., 1973), and the definition of predictability as identical with the presence or absence of a signal preceding a regularly occurring aversive stimulus was criticized (Bassett et al., 1973). Indeed, the predictability of a stressor in terms of time has been proposed as an important dimension of its consequences. For example, repeated stress applied varying the interval between each exposure (i.e., irregular) induced greater and more persistent increase in plasma corticosterone, fatty acid, and glucose levels than regular exposure (Bassett et al., 1973; Quirce et al., 1981; Smith et al., 2013). Increase in anxiety- and depression-like behaviors and somatic changes such as adrenal hypertrophy and reduction in body weight gain were also rather observed following stressor applied irregularly than regularly (Martí and Armario, 1997; Smith et al., 2013), besides some of these effects may be related to stressor intensity (Martí and Armario, 1997). Studies have also used protocols that vary the duration of each stress session, but with constant interval between the sessions (Rockman et al., 1987; Ortiz et al., 2015). Nevertheless, a comparison of the responses induced by this stress paradigm with those induced by a stressor applied regularly has never been investigated.

Studies have also evaluated the predictability by varying the type of stressor. These studies have compared the impact of daily exposure to the same stressor type (i.e., homotypic) vs. the exposure to different aversive stimuli (i.e., heterotypic). Typically, these studies have compared the effects of the RRS applied in a predictable schedule vs. the CVS, which is a widely used paradigm that involves daily exposure of rodents to different stressors at unpredictable times (Willnér, 2005; Grippo, 2009; Frisbee et al., 2015). In this regard, the CVS has been demonstrated to induce more severe somatic changes such as adrenal hypertrophy and thymus involution (Magariños and McEwen, 1995; Zucchi et al., 2009; Flak et al., 2012; Kopp et al., 2013; Duarte et al., 2015a), which is possibly related to an increase in baseline HPA axis activity observed mainly following exposure to heterotypic stressors (Magariños and McEwen, 1995; Marin et al., 2007). Increase in anxiety- and depression-like behaviors was also more severe following exposure to CVS than to RRS (Haile et al., 2001; Pastor-Ciurana et al., 2014; Yoon et al., 2014; Zhu et al., 2014; Gao et al., 2016). Although some studies report that CVS induces increased reduction in body weight gain (Marin et al., 2007; Gao et al., 2016), several studies have demonstrated that homotypic and heterotypic stressors similarly affect this parameter (Magariños and McEwen, 1995; Vyas et al., 2002; Flak et al., 2012; Yoon et al., 2014; Duarte et al., 2015a).

The lesser impact of homotypic stressors in neuroendocrine, behavioral, and some somatic parameters is possibly related to a habituation process upon repeated exposure to the same stressor, which is reduced during exposure to heterotypic stressors (Herman, 2013). This habituation process is mainly evidenced by a progressive reduction in HPA axis activation (Grissom and Bhatnagar, 2009). Indeed, adaptation to chronic stress determined by habituation of responses upon repeated stress exposure has been proposed, which limits the long-term impact of stress (Grissom and Bhatnagar, 2009; Herman, 2013). In this regard, the habituation process has been demonstrated to be impaired when homotypic stressors are applied irregularly (De Boer et al., 1989; Martí and Armario, 1997; Smith et al., 2013), which is in line with evidence discussed above wherein stressors applied irregularly induce more severe responses.

### Influence of predictability in cardiovascular responses to stress

Cardiovascular and autonomic changes have been reported following exposure to both predictable and unpredictable stressors. However, few studies to date have investigated the influence of predictability by directly comparing cardiovascular and autonomic changes induced by predictable vs. unpredictable stress protocols. De Boer et al. (1989) initially demonstrated that noise applied regularly, but not irregularly, increased plasma noradrenaline concentration, whereas noradrenaline response to noise was decreased in animals subjected to irregular protocol. Plasma adrenaline response to noise was reduced by both regular and irregular protocols (De Boer et al., 1989). These results do not support an influence of predictability in the sympatho-adrenomedullary response to stress. However, a possible influence of regular vs. irregular aversive stimuli on cardiovascular parameters, such as blood pressure and HR, has never been evaluated. Indeed, an impact of predictable vs. unpredictable stressors in cardiovascular function was addressed only recently. In this regard, Duarte et al. (2015a) compared the effect of the homotypic stressor RRS (predictable) vs. the heterotypic stressor CVS (unpredictable) on the baseline values of arterial pressure and HR, baroreflex function, and cardiac autonomic activity. They observed that both chronic stressors increased the baseline arterial pressure values (Duarte et al., 2015a). However, increase in baseline HR values and cardiac sympathetic activity as well as impaired baroreflex function was observed only in animals subjected to CVS (Duarte et al., 2015a). Although, these results provide evidence of a more severe impact of unpredictable vs. predictable stressors in cardiovascular function and autonomic activity, relevant cardiovascular changes were also detected following exposure to the predictable stressor RRS.

As stated above, adaptation to chronic stress determined by habituation of physiological responses upon repeated exposure to the same stressor has been proposed, which limits its long-term impact (Grissom and Bhatnagar, 2009; Herman, 2013). This idea has been supported mainly by consistent findings of habituation of HPA axis activation (Grissom and Bhatnagar, 2009). Table 3 summarizes the studies that compared cardiovascular and autonomic responses during an acute stress session and after repeated exposure to the stressor. We note that studies have not consistently demonstrated a habituation of cardiovascular responses upon repeated exposure to a stressor (Table 3). For example, some findings indicated a decrease in pressor and tachycardiac response upon repeated exposure to restraint stress (Chen and Herbert, 1995; Bechtold et al., 2009), but several other studies reported similar cardiovascular responses to this stressor during both acute and repeated exposures (McDougall et al., 2000, 2005; Conti et al., 2001; Daubert et al., 2012). Analysis of cardiovascular and autonomic responses during repeated exposure to social defeat has also identified inconsistent results. Indeed, some results evidenced habituation (Adams et al., 1987; Chung et al., 1999; Costoli et al., 2004), whereas several other studies reported similar responses during repeated exposure to social defeat (Adams and Blizard, 1986; Meehan et al., 1995; Sgoifo et al., 2001, 2002; Carnevali et al., 2012, 2013b). Regardless of inconsistency of the results, evidence that cardiovascular responses do not readily habituate indicates a reduced process of adaptation (Grissom and Bhatnagar, 2009; Herman, 2013), which supports the findings of Duarte et al. (2015a) and other researchers (see above sections) demonstrating significant cardiovascular dysfunctions following exposure to predictable stressors (e.g., RRS and repeated social defeat).

**Table 3:** **Studies comparing arterial pressure, heart rate (HR), and autonomic responses during an acute session of stress and after repeated exposure to the stressor**.

**Specie/Strain**	**Age/Gender**	**Stressor and Protocol length**	**Arterial pressure**	**HR**	**Autonomic activity**	**References**
WT Groningen rat	10 weeks/male	Social defeat (12 episodes/75 min in 25 days)	–	Tachycardia =	–	Carnevali et al., 2013b
5-HT1A KO mice WT mice	12 weeks/male	Social defeat (cohabited with resident, 5 defeat episodes in 15 days)	–	Tachycardia =	RMMSD =	Carnevali et al., 2012
Sprague-Dawley rat	275–300 g/male	Restraint 60 min/session 5 sessions	Pressor response =	Tachycardia =	–	Daubert et al., 2012
**WKY rat BHR rat**	**WKY – 414 g BHR – 385 g male**	**Restraint 1**–**2 h/session 5**–**7 sessions/week 21**–**25 days**	**Pressor response** ↓ **(both strain)**	**Tachycardia** ↓ **(both strain)**	–	**Bechtold et al., 2009**
WKY rat SHR rat Sprague-Dawley rat	15–16 weeks/male	Handling ~30s/session 10 days Air-jet stress 1h/day 10 days Restraint 1h/day 10 days	Pressor response =	Tachycardia =	–	McDougall et al., 2005
**Swiss CD-1/mice**	**12 weeks/male**	**Social defeat (cohabited with resident, 5**–**15 min agonistic interaction/day) 15 days**	–	**Tachycardia** ↓ **(only post-interaction)**	–	**Bartolomucci et al., 2003**
**Swiss CD-1/mice**	**12 weeks/male**	**Social defeat (cohabited with resident, 5**–**15 min agonistic interaction/day) 15 days**	–	**RR interval** ↓	**SD**_RR_ **and r-MSSD** ↓	**Costoli et al., 2004**
WT Groningen rat	5 months/male	Social defeat 10 days Open field 10 days	–	RR interval = Cardiac arrhythmia =	Cardiac autonomic response- No habituation	Sgoifo et al., 2002
WKY/male	12 weeks	Restraint 20 min/day 5 days	Pressor response =	Tachycardia =	–	Conti et al., 2001
WT Groningen rat	5 months/male	Social defeat Social victory 15min/session 10 sessions	–	**Victory RR interval and cardiac arrhythmias** ↓ Defeat RR and cardiac arrhythmias =	Victory SDRR and r-MSSD = Defeat SDRR and r-MSSD =	**Sgoifo et al., 2001**
**WKY rat SHR rat**	**15**–**16 weeks male**	**Restraint 1 h/day 10 sessions**	**WKY** = **SHR** =	**WKY** = **SHR** ↓ **(duration)**	–	**McDougall et al., 2000**
**Lister hooded rat**	**180 g/male**	**Social defeat 20 min/session (10 min interaction without contact**+**10 min direct interaction) 10 days (experiments light off)**	–	**Tachycardia – partial habituation**	–	**Chung et al., 1999**
Wistar rat	Adult/male	Acoustic stimuli 5 stimuli in 60 s	Pressor response =	Tachycardia followed by bradycardia =	–	Blanc et al., 1997
**Lister Hooded rats**	**250 g/male**	**Restraint 1 h/day 10 days**	–	**Tachycardia** ↓	–	**Chen and Herbert, 1995**
Long Evans rat	350–400 g male	Social defeat Submission + 55 min protected contact (*experiments light off*)	Pressor response =	Tachycardia =	–	Meehan et al., 1995
**S/JR rat**	**70**–**80 days male**	**Social defeat 25 min/session 6 sessions Footshock 25min/session(intermittent) 6 sessions**	**Defeat – habituation of depressor response Footshock – habituation pressor response Direct measurement over 2 sessions- Defeat – facilitation depressor response in second session Footshock –Non-significant response**	**Defeat – non-significant effect Footshock** = **over days Direct measurement over 2 sessions- Defeat – habituation Footshock - habituation**	–	Adams et al., 1987^*^
Dahl-S+salt Dahl-R+salt	70–80 days (adult)	Social Defeat (25 min) 6 sessions	S/JR depressor response = R/JR Non-significant response	S/JR Non-significant response R/JR Tachycardia =	–	Adams and Blizard, 1986^*^

BHR, borderline hypertensive rat; Dahl-S, Dahl salt-sensitive rats; Dahl-R, Dahl salt-resistant rats; KO, knockout mice; SHR, spontaneously hypertensive rats; WKY, Wistar-Kyoto rat.

Bold indicates studies that identified reduction of cardiovascular and/or autonomic responses upon repeated exposure to the same stressor (i.e., habituation).

* Tail-cuff method of cardiovascular recording.

Reasons for the discrepancy in findings of habituation of cardiovascular responses are unclear, but may be the result of methodological differences. For example, the protocol of one of the studies that identified habituation included some stress-free days (i.e., animals were subjected to restraint stress 5–7 days/week for 21–25 days; Bechtold et al., 2009), which is different from the studies that did not identify habituation to restraint stress wherein animals were continuously subjected to stress. In this regard, stress-free periods between periods of repeated stress exposure has been proposed to facilitate the habituation process (Grissom and Bhatnagar, 2009). Differences between species and strains may also contribute. For example, more consistent evidence of habituation to social defeat was observed in mice (Bartolomucci et al., 2003; Costoli et al., 2004), whereas similar responses upon repeated exposure to social defeat was observed in rats (Adams and Blizard, 1986; Meehan et al., 1995; Sgoifo et al., 2001, 2002; Carnevali et al., 2013b). A study using rats reported habituation upon repeated exposure to social defeat (Chung et al., 1999), whereas another report in mice did not identify habituation (Carnevali et al., 2012). However, strains of mice and rats used in these studies were different from those wherein a habituation process was observed in mice rather than in rats (Table 3), indicating that the strain may also affect the habituation process. Nevertheless, further studies are necessary to better address the habituation of the cardiovascular responses upon repeated exposure to the same stressor.

## Concluding remarks

A significant increase in research using animal models on the impact of emotional stress in cardiovascular function and autonomic activity has occurred in last years. These studies have provided evidence that the impact of stress on the cardiovascular function is determined by stressor stimulus characteristics, such as chronicity and predictability. Regarding chronicity, changes in cardiovascular function and autonomic activity are well-documented during acute stress sessions. These responses constitute important short-term adaptive mechanisms, maintaining homeostasis and ensuring survival. However, enduring autonomic imbalance and cardiovascular dysfunctions are provoked mainly by long-term exposure to stressful events (i.e., chronic stress). In this sense, studies have provided consistent results regarding the effect of chronic emotional stress in baseline HR values, autonomic activity, and baroreflex function. Nevertheless, the chronic stress paradigm seems to be an important factor that determines the pattern of changes in these parameters. A detailed analysis of the effects of chronic stress in arterial pressure reveals inconsistent results; hence, an animal model of stress-induced hypertension is still missing.

Analysis of neuroendocrine and behavioral responses to stress has clearly demonstrated an influence of predictability of stressor in its effects. Nevertheless, only a limited number of studies compared the cardiovascular and autonomic changes following exposure to predictable vs. unpredictable stressors. These studies have not demonstrated a clear influence of predictability in cardiovascular dysfunctions induced by chronic stress. However, a possible influence of predictability in stress-induced cardiovascular complications is only beginning to be investigated, and a number of issues need to be addressed before more conclusive analysis can be performed. For example, adaptation to chronic stress determined by habituation of physiological responses upon repeated exposure to the same stressor has been proposed (Grissom and Bhatnagar, 2009; Herman, 2013), which explains the more severe impact of homotypic vs. heterotypic stressors in HPA axis activity, behavioral responses, and somatic parameters. However, habituation of the cardiovascular and autonomic responses is still a matter of debate. Furthermore, evaluation of cardiovascular changes following exposure to homotypic stressors applied regularly vs. irregularly is a relevant unvalued aspect in determining the influence of predictability, because this approach compares responses with an aversive stimulus of similar intensity applied at predictable vs. unpredictable schedules. Therefore, further studies are necessary to elucidate the influence of predictability of stressor in its cardiovascular responses.

## Author contributions

CC designed, drafted, and revised the manuscript; and prepared the figures and tables.

### Conflict of interest statement

The author declares that the research was conducted in the absence of any commercial or financial relationships that could be construed as a potential conflict of interest.
